# Novel Preoperative Carbohydrate Drinks Versus Commercial Syrup-Based Drinks on Gastric Emptying, Glycemic Responses, and Fasting Discomfort: A Pilot Randomized Crossover Trial

**DOI:** 10.3390/nu17132131

**Published:** 2025-06-27

**Authors:** Chaitong Churuangsuk, Khanin Khanungwanitkul, Anukoon Kaewborisutsakul, Chanatthee Kitsiripant, Athithan Rattanaburi, Onnicha Suntornlohanakul, Krit Charupanit, Thammasin Ingviya, Utcharee Intusoma, Panupong Puttarak

**Affiliations:** 1 Clinical Nutrition and Obesity Medicine Unit, Department of Internal Medicine, Faculty of Medicine, Prince of Songkla University, Songkhla 90110, Thailand; chaitong.c@psu.ac.th; 2 Department of Radiology, Faculty of Medicine, Prince of Songkla University, Songkhla 90110, Thailand; 3 Neurological Surgery Unit, Department of Surgery, Faculty of Medicine, Prince of Songkla University, Songkhla 90110, Thailand; anukoonkaew@gmail.com; 4 Department of Anesthesiology, Faculty of Medicine, Prince of Songkla University, Songkhla 90110, Thailand; chanat.k@gmail.com; 5 Division of Gynecologic Oncology, Department of Obstetrics and Gynecology, Faculty of Medicine, Prince of Songkla University, Songkhla 90110, Thailand; r_athithan123@hotmail.com; 6 Endocrinology and Metabolism Unit, Department of Internal Medicine, Faculty of Medicine, Prince of Songkla University, Songkhla 90110, Thailand; onnicha.sun@gmail.com; 7 Department of Biomedical Sciences and Biomedical Engineering, Faculty of Medicine, Prince of Songkla University, Songkhla 90110, Thailand; krit.ch@psu.ac.th; 8 Department of Family Medicine and Preventive Medicine, Faculty of Medicine, Prince of Songkla University, Songkhla 90110, Thailand; thammasin.i@psu.ac.th; 9 Department of Pediatrics, Faculty of Medicine, Prince of Songkhla University, Songkhla 90110, Thailand; utchareei@yahoo.com; 10 Department of Pharmacognosy and Pharmaceutical Botany, Faculty of Pharmaceutical Sciences, Prince of Songkla University, Songkhla 90110, Thailand; panupong.p@psu.ac.th; 11 Phytomedicine and Pharmaceutical Biotechnology Research Center, Prince of Songkla University, Songkhla 90110, Thailand

**Keywords:** preoperative carbohydrate loading, gastric emptying, patient reported outcome, enhanced recovery after surgery (ERAS), glycemic response, perioperative care

## Abstract

**Background**: Enhanced Recovery After Surgery (ERAS) guidelines recommend preoperative carbohydrate loading; however, local hospitals often use syrup concentrate sweet drinks rather than specialized carbohydrate formulations. We compared gastric emptying, glycemic response, and fasting discomfort of a novel carbohydrate drink versus syrup concentrate sweet drinks. **Methods**: In this pilot randomized, crossover trial at Prince of Songkla University Hospital, Thailand, 16 healthy volunteers received three interventions with a 1-week washout period: novel carbohydrate drink 400 mL (C400), novel carbohydrate drink 250 mL (C250), and syrup sweet drink 250 mL (SYR). The primary outcome was gastric antral cross-sectional area (CSA) measured using ultrasonography at baseline, 10, 60, 120, and 180 min post-ingestion. Secondary outcomes included glycemic response and visual analog scales for thirst and hunger. **Results**: All drinks showed comparable gastric CSA, peaking at 10 min (5.5–6.5 cm^2^, *p* < 0.01) and returning to baseline by 120 min. Novel carbohydrate drinks produced higher glucose peaks at 60 min (C400: 147.4 mg/dL [28.0]; C250: 148.7 mg/dL [21.7]) than SYR (123.1 mg/dL [22.4], *p* = 0.006) with sustained elevation through 120 min. All drinks similarly reduced thirst and mouth dryness scores at 60 min (*p* < 0.05), though hunger increased progressively after 120 min across all groups. **Conclusions**: Both 400 mL and 250 mL volumes of novel carbohydrate drinks demonstrated safe gastric emptying profiles comparable to syrup concentrate sweet drinks while providing more sustained glycemic responses.

## 1. Introduction

Prolonged preoperative fasting has been increasingly recognized as detrimental to surgical outcomes, leading to adverse physiological consequences, including increased insulin resistance, protein catabolism, and patient discomfort [1,2,3,4,5]. The Enhanced Recovery After Surgery (ERAS^®^) protocols now recommend consuming 50 g of complex carbohydrates in 400 mL of clear fluid 2–3 h before anesthetic induction, a practice incorporated into many perioperative guidelines [6,7,8,9,10,11]. These recommendations represent a paradigm shift from traditional “nil per mouth” after midnight practices, which persist in many healthcare settings globally [6,7,8,9,10,11,12,13].

Despite robust evidence supporting preoperative carbohydrate loading in Western populations [1,14,15,16], a critical gap exists regarding its safety and efficacy in Asian populations. This gap is particularly relevant given documented ethnic differences in gastric emptying physiology, body composition, insulin sensitivity, and healthcare resources [17,18,19,20,21]. These variations suggest that Western-derived protocols may require adaptation for optimal safety and efficacy in Asian surgical patients.

The implementation of evidence-based preoperative carbohydrate loading in Asia faces practical challenges. Standard maltodextrin-based formulations (12.5% concentration, osmolality 285–290 mOsm/kg) used in Western studies are often unavailable or high cost, leading many Asian hospitals to substitute locally available syrup concentrate drinks or fruit juices [22,23,24]. This pragmatic adaptation raises important safety concerns as these alternatives differ substantially from evidence-based formulations—containing primarily simple sugars rather than complex carbohydrates, with higher osmolality (>350 mOsm/kg) that may delay gastric emptying [23,24,25,26].

To address these scientific and clinical gaps, we developed a novel carbohydrate drink specifically formulated for preoperative use in Asian populations, considering regional physiological characteristics and local taste preferences. This effort serves as a pioneering model for bridging the global–local evidence divide, providing a solution designed to be culturally and economically appropriate, thereby increasing the likelihood of successful implementation.

Our primary objective was to establish the gastric emptying profile of the novel formulation at two volumes (400 mL and 250 mL) compared to commercially available syrup-based drinks (250 mL) currently used in several Asian hospitals, using real-time ultrasonographic assessment. Secondary objectives included evaluating postprandial glycemic responses and patient-reported fasting discomfort measures.

We hypothesized that the novel formulation would demonstrate non-inferior gastric emptying profiles compared to syrup-based drinks, while providing more sustained glycemic response. By establishing safety and efficacy data specific to an Asian population, this study contributes essential evidence for adapting international ERAS protocols across diverse global populations while addressing region-specific implementation barriers.

## 2. Materials and Methods

### 2.1. Study Design and Ethics

This acute physiological study was designed as a prospective, double-blind, randomized, crossover trial. The study protocol was approved by the Human Research Ethics Committee of Prince of Songkla University (REC.65-137-14-1) and registered with the Thai Clinical Trial Registry (TCTR20220323001, first trial registration date 23 March 2022). Written informed consent was obtained from all participants. The study was conducted in accordance with the Declaration of Helsinki and Good Clinical Practice guidelines.

### 2.2. Participant Inclusion and Exclusion Criteria

A total of 16 healthy volunteers were recruited. Inclusion criteria were healthy adults aged 18 to 60 years. Exclusion criteria included any history, symptoms, or signs of upper gastrointestinal disorders, diabetes mellitus (any type), malignancy, kidney or liver diseases, and current use of medications for chronic diseases. Participants were also excluded if they were currently using any medications known to affect gastrointestinal motility (e.g., prokinetics, opioids, anticholinergics) or had any history of gastric surgery that could alter normal gastric emptying.

### 2.3. Test Beverages

Three test beverages were studied:

1. Novel carbohydrate drink 400 mL (C400)
2. Novel carbohydrate drink 250 mL (C250)
3. Syrup concentrates 250 mL (SYR)

The novel carbohydrate drink was developed and manufactured under Good Manufacturing Practice standards specifically for preoperative use. The formulation contains 50 g complex carbohydrates, primarily maltodextrin (50%), sucrose (19%), isomalt (19%), premixed vitamins (0.1%), and ginger flavor (11.9%). Chemical properties include pH 6.51, osmolality 207 mOsm/kg, and energy content of 200 calories per serving.

The syrup concentrate sweet drink was prepared using Hale’s Blue Boy flavored syrup, with 60 mL of syrup diluted to a final volume of 250 mL to achieve 50 g of carbohydrate (pH 5.71, osmolality 365 mOsm/kg).

**Figure 1:**
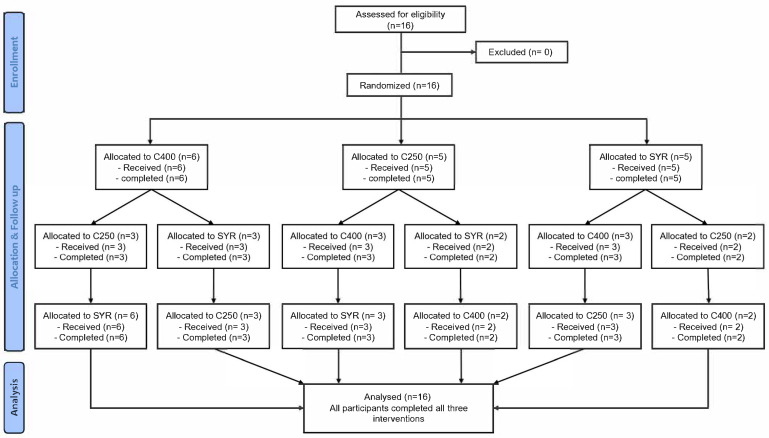
Consort flow diagram of the study.

Each participant underwent three separate study sessions with a minimum 1-week washout period between sessions (Figure 1).

### 2.4. Patient and Public Involvement

Patients or members of the public were not involved in the design, conduct, reporting, or dissemination plans of this research. However, feedback from previous patients regarding preoperative drink preferences and volumes was considered during the development of the novel carbohydrate formulation. The choice to include ginger was influenced by its cultural familiarity and acceptance in the Thai population. Future implementation studies would benefit from greater patient and public involvement, particularly regarding preferences for drink volume and flavor options.

### 2.5. Randomization and Blinding

Block randomization was used to determine the sequence of drink administration. We constructed a random-number table before study initiation and prepared sequentially numbered, sealed, opaque envelopes containing beverage sequence assignments. On each study day, a research assistant not involved in participant evaluation opened the corresponding envelope and prepared the beverages in identical packaging. The radiologist performing ultrasound examinations and the investigators involved in data collection were blinded to beverage assignments.

A minimum of a 1-week washout period was set to minimize carryover effects. This duration is generally considered sufficient for the complete elimination of residual effects from a single dose of carbohydrate and its metabolic byproducts.

### 2.6. Study Protocol

Participants were instructed to fast overnight for at least 8 h before each study session, with a minimum one-week washout period between sessions. They were asked to abstain from alcohol, caffeine, and strenuous exercise the day before the examination. On study day, baseline measurements were obtained at 8:30 a.m., followed by the consumption of the assigned beverage within 5 min.

### 2.7. Primary Outcome

Gastric emptying was evaluated by measuring the antral cross-sectional area (CSA) using bedside ultrasonography (Samsung Medison Co., Ltd., Seoul, Republic of Korea, Model SONOACE R7). All measurements were performed by the same experienced radiologist. Participants were positioned supine. The gastric antrum was identified in the sagittal plane passing through the aorta. Longitudinal (D1) and anteroposterior (D2) diameters were measured at the level of the abdominal aorta and left liver lobe. Three measurements were taken at each time point to calculate the mean of the D1 and D2 values. Then, the antral CSA was calculated using the formula: A = π × (D1mean × D2mean)/4 [18,27]. Measurements were obtained at five time points: baseline (0 min) and at 10, 60, 120, and 180 min post-ingestion, with a window of ±5 min at each time point.

### 2.8. Secondary Outcomes

Postprandial glycemic response was assessed using point-of-care capillary blood glucose testing. Measurements were obtained at baseline and at 60, 120, and 180 min after beverage consumption (±5 min for each time point).

Subjective fasting discomfort was evaluated using standardized 100 mm visual analog scales (VAS). Participants marked their level of discomfort on three separate scales assessing thirst (0 = not thirsty at all, 100 = extremely thirsty), mouth dryness (0 = not dry at all, 100 = extremely dry), and hunger (0 = not hungry at all, 100 = extremely hungry). VAS assessments were conducted at the same time points as blood glucose measurements.

No changes were made to the trial outcomes after the trial commenced. The primary and secondary outcomes remained as specified in the original protocol.

### 2.9. Sample Size Calculation

This study was designed to assess gastric antral CSA before and after carbohydrate drink consumption, with the hypothesis that the gastric antral CSA would return to baseline levels after consuming the carbohydrate drink. Therefore, a one-sample equivalence design was used to calculate sample size.

Based on Jian et al.’s study [28] of 400 mL carbohydrate drinks, which reported baseline gastric antral CSA of 306 mm^2^ (SD 220) and 300 mm^2^ at 2 h post-consumption, we used their observed standard deviation for our power calculation. In addition, previous research has demonstrated that the gastric antral CSA of 900–1000 mm^2^ measured using ultrasonography does not increase aspiration risk in healthy individuals [13].

For one-sample equivalence design, we set a non-inferiority margin (δ) of 150 mm^2^, meaning that if post-consumption gastric CSA remained within 150 mm^2^ of baseline values, this would be considered clinically equivalent to complete gastric emptying. For context, using Jian et al.’s baseline CSA of 306 mm^2^, a post-consumption CSA up to 456 mm^2^ (306 + 150) would still be considered safe and not associated with increased aspiration risk. This margin was chosen as a conservative threshold well below the 900–1000 mm^2^ range that ultrasonographic studies have identified as the upper safety limit for aspiration risk in healthy individuals.

With α = 0.05, power = 80%, expected mean difference of 6 mm^2^ (based on Jian et al.’s findings), standard deviation of 220 mm^2^, and non-inferiority margin of 150 mm^2^, we calculated a required sample size of 15 participants.

For the comparison of gastric emptying over time between groups, we performed a longitudinal linear model slope power calculation based on Jian et al.’s data [28]. Their study showed a delta of 29 and a sigma^2^ of 10, indicating a minimum requirement of six participants per group. Since the primary objective required a larger sample size of 15 subjects, this was sufficient to address both study aims.

### 2.10. Statistical Analysis

All statistical analyses were performed using R software (version 4.0.2; R Foundation for Statistical Computing, Vienna, Austria). Normality of distribution was assessed using the Shapiro–Wilk test for all continuous variables. Descriptive statistics were presented as means with SD for normally distributed variables and medians with interquartile ranges [IQR] for non-normally distributed variables. Categorical variables were summarized as frequencies and percentages.

Between-group comparisons were conducted using one-way analysis of variance (ANOVA) for normally distributed variables, followed by Tukey’s honest significant difference test for post-hoc pairwise comparisons when significant differences were detected. For non-normally distributed variables, the Kruskal–Wallis test was employed. Within-group comparisons across time points were performed using paired *t*-tests for normally distributed data and Wilcoxon signed-rank tests for non-normally distributed data.

The area under the curve (AUC) was calculated using the trapezoidal method for each participant’s time-series data. Between-group differences in AUC were evaluated using one-way ANOVA or the Kruskal–Wallis test, depending on data distribution. When significant differences were identified, post-hoc analyses were conducted using Tukey’s HSD test or Dunn’s test, as appropriate. Statistical significance was set at *p* < 0.05, and all tests were two-tailed. For post-hoc multiple comparison, *p* < 0.01 was considered significant.

## 3. Results

### 3.1. Study Participants

A total of 16 participants (15 female) were screened and enrolled (from 6 May 2022 to 19 June 2022) in the study. All participants completed all three intervention sessions with no dropouts. No early stopping was required as no safety concerns emerged during the study period. The mean age was 33.7 years (SD 8.1), mean body weight 61.2 kg (SD 14.2), and mean body mass index 24.0 kg/m^2^ (SD 5.1; Table 1).

### 3.2. Effect on CSA of the Gastric Antrum

According to the ERAS guidelines, a novel carbohydrate drink with a volume of 400 mL was administered. Additionally, 250 mL of a novel carbohydrate drink and 250 mL of Hell’s Blue Boy syrup sweet drink were also consumed, a volume representing the current practice in this region.

**Figure 2:**
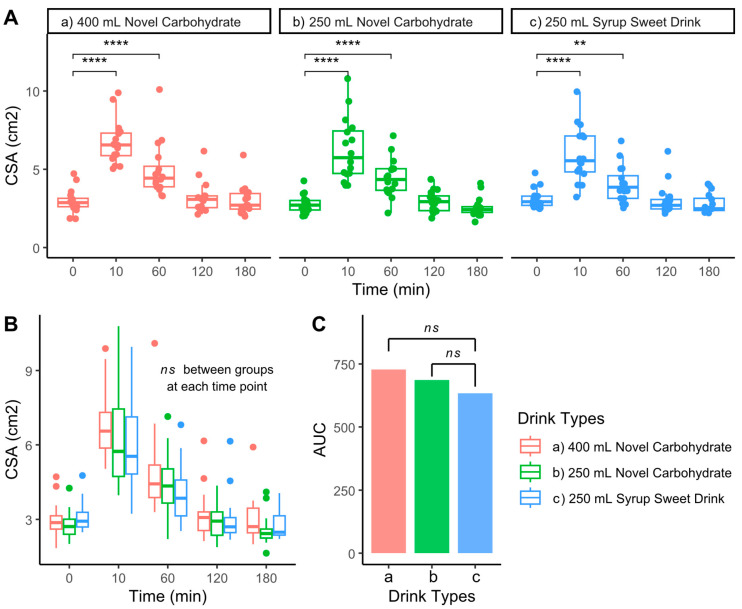
Comparison of gastric cross-sectional area (CSA) response to different carbohydrate drinks. (**A**) Time course of CSA measurements following consumption of 400 mL novel carbohydrate drink, 250 mL novel carbohydrate drink, and 250 mL syrup sweet drink. (**B**) Overlaid comparison of CSA responses between drink types. (**C**) Area under the curve (AUC) analysis comparing total CSA response across drink types. **** *p* < 0.0001, ** *p* < 0.01, ns: not significant.

Gastric CSA was measured following consumption of three different carbohydrate drinks (Figure 2A and Table A1). All beverages induced significant increases in CSA from baseline (2.7–2.9 cm^2^) to peak values (5.7–6.6 cm^2^, *p* < 0.01–0.0001) at 10 min, followed by gradual declines to baseline level at 120 and 180 min post-ingestion. Despite differences in volume and formulation, direct comparisons revealed no significant differences in CSA between drinks at any time point (Figure 2B).

Furthermore, an analysis of the total response, as measured by AUC, showed comparable values across all three drinks with no statistically significant differences between conditions (Figure 2C). These findings suggest that gastric accommodation and emptying patterns were similar regardless of the drink volume or carbohydrate formulation used.

### 3.3. Effects on Postprandial Glycemic Response

**Figure 3:**
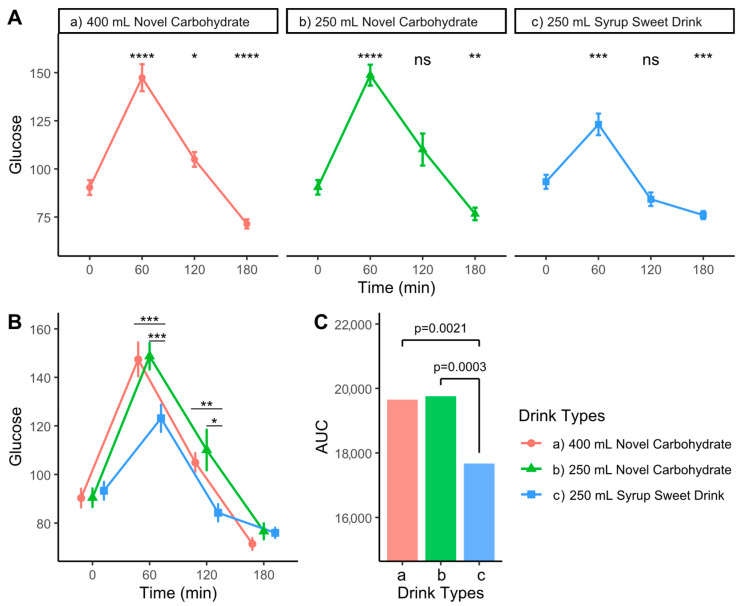
Glycemic responses to different carbohydrate drinks: (**A**) Time courses of blood glucose following consumption of 400 mL novel carbohydrate drink, 250 mL novel carbohydrate drink, and 250 mL syrup sweet drink. Statistical significance from baseline is indicated by asterisks. (**B**) Overlaid comparison of glucose responses between drink types, with asterisks indicating significant differences between groups at specific time points. (**C**) Area under the curve (AUC) analysis comparing total glucose response across drink types. **** *p* < 0.0001, *** *p* < 0.001, ** *p* < 0.01, * *p* < 0.05, ns: not significant. Error bars represent the standard error of the mean.

Capillary blood glucose levels were measured following consumption of three different carbohydrate drinks (Figure 3A and Table A1). All beverages induced significant increases in blood glucose, peaking at 60 min post-consumption (C400 and C250: 147.4 (28.0) and 148.7 (21.7) mg/dL, *p* < 0.0001; SYR: 123.1 (22.4) mg/dL, *p* < 0.001). Direct comparison between drinks revealed significantly higher glucose responses for both novel carbohydrate drinks compared to the syrup sweet drink at 60 min (*p* < 0.001) and 120 min (*p* < 0.01–0.05; Figure 3B). The analysis of the total glycemic response showed significantly higher AUC values for both the 400 mL (*p* = 0.0021) and 250 mL novel carbohydrate drinks (*p* = 0.0003) compared to the syrup sweet drink (Figure 3C), demonstrating their superior ability to sustain elevated blood glucose levels during the preoperative fasting period.

### 3.4. Patient-Reported Outcomes on Fasting Discomfort

**Figure 4:**
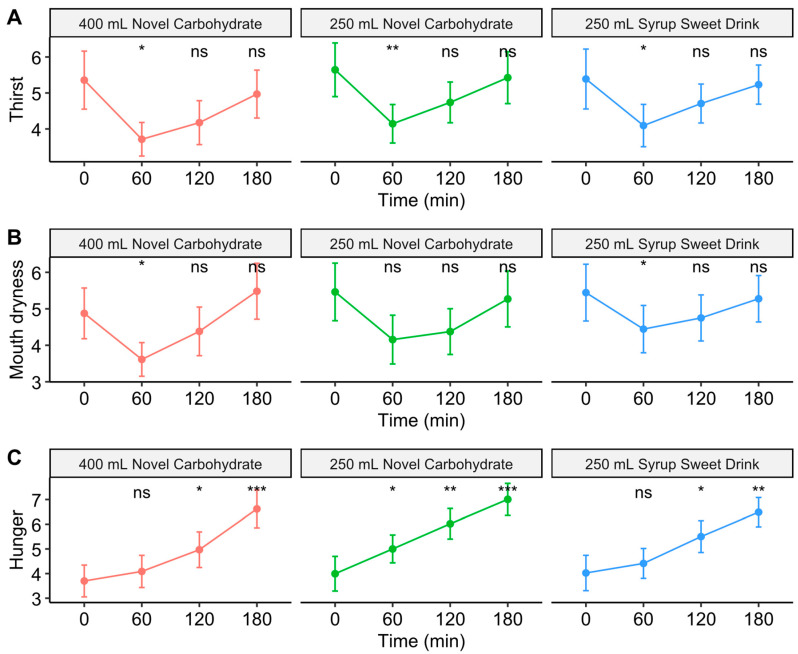
Visual analogue scale (VAS, cm) ratings of thirst, mouth dryness, and hunger following carbohydrate drink consumption. (**A**) Time course of thirst VAS ratings, (**B**) mouth dryness VAS ratings, and (**C**) hunger VAS ratings after consuming 400 mL novel carbohydrate drink, 250 mL novel carbohydrate drink, and 250 mL syrup sweet drink. Statistical significance from baseline is indicated by asterisks. *** *p* < 0.001, ** *p* < 0.01, * *p* < 0.05, ns: not significant. Error bars represent the standard error of the mean.

Patient-reported discomfort was assessed using a 100 mm VAS. Thirst scores decreased from baseline at 60 min for all drinks (*p* < 0.05–0.01), followed by gradual increases toward baseline levels (Figure 4A and Table A1). Similarly, mouth dryness scores decreased significantly at 60 min for the 400 mL novel carbohydrate drinks and syrup sweet drinks (*p* < 0.05), with no significant changes observed for the 250 mL novel carbohydrate drinks (Figure 4B). Hunger ratings increased progressively over time for all drinks, with significant increases from baseline observed from 120 min onwards (*p* < 0.05–0.001; Figure 4C). The pattern of these subjective VAS responses was similar across all drink types, suggesting comparable effects on thirst, mouth dryness, and hunger regardless of drink volume or carbohydrate formulation.

### 3.5. Safety and Adverse Events

No adverse events or safety concerns were reported during any of the study sessions. All participants tolerated the drinks well, with no episodes of nausea, vomiting, or abdominal discomfort reported.

## 4. Discussion

Our study provides important insights into the gastric emptying profiles and metabolic responses of different preoperative carbohydrate formulations in a Southeast Asian population. The ultrasonographic assessment revealed that all three beverage formulations showed similar patterns of gastric emptying, with CSA returning to baseline levels within 120 min, indicating the empty stomach as CSA < 3.4 cm^2^ [18]. This finding is particularly significant for the 400 mL volume, which aligns with recent ERAS guidelines supporting the safety of larger preoperative fluid volumes [6,7,8,9,12]. Similar to findings by Kitsiripant et al. (2024), who demonstrated safe gastric emptying of 400 mL carbohydrate solutions in individuals with obesity [23], our results support the safety of this volume in normal-weight Southeast Asian individuals. The observed CSA values remained well below the 10 cm^2^ threshold associated with increased aspiration risk, consistent with established safety parameters [13,27].

A notable finding was a more sustained glycemic response achieved with the novel carbohydrate formulation compared to the syrup-based drink. Both volumes of the novel drink maintained significantly higher blood glucose levels at 60 and 120 min post-consumption, and as represented by higher AUC values. This sustained glycemic response is particularly relevant in the context of preoperative metabolic optimization [4,29], as previous studies have demonstrated that maintaining adequate preoperative glucose levels can reduce postoperative insulin resistance [14,15,30,31]. The difference in glycemic response despite similar gastric emptying profiles suggests that the complex carbohydrate composition of the novel drink offers metabolic advantages over simple sugar-based solutions.

The detrimental effects of prolonged preoperative fasting on metabolic homeostasis are well-documented, with studies showing increased insulin resistance and accelerated protein catabolism [14,31]. While early attempts to address these issues focused on intravenous glucose administration, this approach presents several practical challenges. Although intravenous carbohydrate loading can improve nitrogen balance and reduce postoperative insulin resistance compared to fasting [29], it may require concurrent insulin administration, intensive glucose monitoring, and carries risks of fluid overload. Moreover, intravenous administration fails to address patient comfort issues such as thirst and hunger [30]. Our study supports oral carbohydrate administration as a more physiological and practical alternative, demonstrating that the novel carbohydrate drink not only achieves effective gastric emptying but also provides sustained glycemic response while improving patient comfort measures. This aligns with current ERAS principles, emphasizing the importance of oral intake for both metabolic optimization and patient well-being [3,32].

The findings have particular relevance for ERAS protocol implementation in Southeast Asian healthcare settings. While previous ERAS guidelines were primarily based on Western populations using specialized preoperative drinks [1,7,11], many hospitals in Thailand (and neighboring countries) rely on locally available syrup drinks as carbohydrate-loading solutions due to limited access to commercial medical preoperative carbohydrate formulations [22,23,24].

Our novel carbohydrate drink offers several advantages for Southeast Asian healthcare settings. The formulation enables lower production costs compared to imported commercial preparations, while local manufacturing capability reduces supply chain dependencies. The comparable gastric emptying profiles between 400 mL and 250 mL volumes also provide flexibility in administration protocols, though the larger volume offers potential advantages in terms of total carbohydrate delivery and hydration status. Additionally, the incorporation of familiar ingredients such as ginger potentially improves patient acceptance in the regional context.

Our assessment of patient comfort parameters revealed comparable effects across all three interventions in reducing thirst and mouth dryness. This aligns with previous studies, such as studies from China or Croatia, reporting similar reductions in fasting discomfort despite different preoperative fluid formulations [28,30,32,33]. The consistent improvement in comfort measures, regardless of drink type or volume, suggests that the primary benefit may be related to fluid provision rather than specific formulations.

Several limitations should be considered. Direct comparison with international commercial formulations is limited by the scarcity of acute physiological studies. Most published research on preoperative carbohydrate drinks focuses on clinical endpoints (length of stay, insulin resistance) rather than real-time gastric emptying assessment. The absence of a water-only or true fasting control group limits our ability to quantify the specific effects of carbohydrate content versus fluid volume alone on gastric emptying and comfort measures. Future studies should include these controls to better isolate the metabolic effects of carbohydrate loading from simple fluid administration.

Our study population consisted predominantly of healthy volunteers, and findings may differ in surgical patients who experience preoperative anxiety and stress, factors known to affect gastric emptying [34]. The homogeneous sample (94% female, narrow BMI range) precludes meaningful subgroup analyses. Future studies should recruit diverse populations with adequate power for stratified analyses by sex, BMI, and age. While our sample size was adequate for detecting differences in gastric emptying, larger implementation studies examining real-world feasibility are needed. Cost-effective analysis comparing the novel drink with existing syrup alternatives was not performed and should be conducted in a larger sample of surgical patients. Finally, the single-center design may limit generalizability across different healthcare settings in Southeast Asia.

We proposed the next steps for advancing this research. A larger clinical trial should be conducted in surgical patients to validate our findings and explore the effectiveness on clinical outcomes. Future studies should also investigate the safety and efficacy of preoperative carbohydrate loading in high-risk surgical patients, including the elderly, those with diabetes, and individuals with obesity.

## 5. Conclusions

In this pilot randomized crossover trial, we demonstrated that a novel carbohydrate drink developed specifically for Asian populations achieved complete gastric emptying within two hours while providing more sustained glycemic response compared to commercially available syrup concentrate sweet drinks. Both 400 mL and 250 mL volumes proved safe, challenging conventional volume restrictions. While our findings suggest potential benefits of the novel carbohydrate drink in healthy volunteers, validation in surgical populations is essential before implementation. These results provide preliminary evidence supporting further investigation rather than immediate clinical adoption.

## Figures and Tables

**Table 1:** Baseline characteristics of study participants.

Characteristics	Values (*n* = 16)
Age, years	33.7 (8.1)
Female sex, n (%)	15 (93.8)
Weight, kg	61.2 (14.2)
Height, cm	1.6 (0.0)
Body mass index, kg/m^2^	24.0 (5.1)
Baseline blood glucose, mg/dL	91.4 (14.8)
Baseline gastric CSA, cm^2^ (Median, [IQR])	2.8 [2.6, 3.3]

Data presented as mean (SD) unless otherwise specified. CSA, cross-sectional area.

## Data Availability

The datasets generated during and/or analyzed in this study are not publicly available due to privacy but can be obtained upon request from the corresponding author.

## Abbreviations

The following abbreviations are used in this manuscript:

ERAS	Enhanced Recovery After Surgery
CSA	Cross-sectional area
VAS	Visual analog scales
SD	Standard deviation
IQR	Inter-quartile range
ANOVA	Analysis of variance
AUC	Area under the curve

## Appendix A

**Table A1:** Gastric cross-sectional area, capillary blood glucose levels, and fasting discomforts (thirst, hunger, and mouth dryness) throughout 180 min with between-group comparisons.

Variables	Time (Minutes)	Novel Carbohydrate Drink 400 mL (*n* = 16)	Novel Carbohydrate Drink 250 mL (*n* = 16)	Syrup Sweet Drink (*n* = 16)	*p*-Value
CSA: median [IQR]	0	2.8 [2.6, 3.2]	2.7 [2.3, 3.3]	2.9 [2.5, 3.3]	0.858
	10	6.6 [6.1, 7.3]	5.9 [4.6, 7.7]	5.7 [4.4, 7.6]	0.356
	60	4.5 [4.0, 5.3]	4.2 [3.7, 5.3]	3.8 [3.3, 4.3]	0.166
	120	3.1 [2.6, 3.5]	3.0 [2.5, 3.4]	2.7 [2.5, 3.0]	0.561
	180	2.8 [2.4, 3.3]	2.5 [2.1, 2.9]	2.7 [2.4, 3.3]	0.242
Glucose (mg/dL)	0	90.3 (15.3)	90.4 (15.1)	93.3 (14.7)	0.816
	60	147.4 (28.0) ^a^	148.7 (21.7) ^a^	123.1 (22.4) ^b^	0.006
	120	104.9 (15.3) ^a^	110.1 (33.3) ^a^	84.2 (14.1) ^b^	0.006
	180	71.4 (9.6)	76.6 (13.0)	76.0 (8.4)	0.323
Thirst Score (cm)	0	5.4 (3.2)	5.6 (3.0)	5.4 (3.3)	0.962
	60	3.7 (1.9)	4.1 (2.1)	4.1 (2.4)	0.823
	120	4.2 (2.4)	4.7 (2.3)	4.7 (2.2)	0.739
	180	5.0 (2.7)	5.4 (2.9)	5.2 (2.2)	0.883
Hunger Score (cm)	0	3.7 (2.6)	4.0 (2.8)	4.0 (2.9)	0.935
	60	4.1 (2.6)	5.0 (2.3)	4.4 (2.4)	0.565
	120	5.0 (2.9)	6.0 (2.5)	5.5 (2.6)	0.539
	180	6.6 (3.1)	7.0 (2.6)	6.5 (2.4)	0.853
Mouth Dryness (cm)	0	4.9 (2.8)	5.5 (3.2)	5.4 (3.1)	0.823
	60	3.6 (1.8)	4.2 (2.7)	4.4 (2.6)	0.613
	120	4.4 (2.7)	4.4 (2.5)	4.8 (2.5)	0.894
	180	5.5 (3.1)	5.3 (3.1)	5.3 (2.5)	0.973

Data are mean (SD) unless indicated otherwise. Different letters indicate significant differences between groups in a post-hoc analysis.
